# Congenital lobar emphysema associated with polysplenia syndrome

**DOI:** 10.4103/0256-4947.70573

**Published:** 2010

**Authors:** Naseer A. Choh, Suhil A. Choh, Majid Jehangir, Bashir A. Naikoo

**Affiliations:** a From the Shri Maharaja Hari Singh Hospital, Kashmir, India; b From the Sher-i-Kashmir Institute of Medical Sciences, Soura, Srinagar, India

## Abstract

Polysplenia, or left isomerism, is a rare heterotaxy syndrome characterized by bilateral bi-lobed lungs, bilateral pulmonary atria, a symmetrical midline liver, and multiple aberrant splenic nodules. We report a case of polysplenia associated with congenital lobar emphysema apart from other typical anomalies. Such an association has not been previously reported. The patient was a young male with progressive exertional breathlessness referred for high resolution CT of the lungs. CT, MRI and echocardiography revealed (in addition to congenital lobar emphysema of right lung) a hemiazygos continuation of the inferior vena cava, a persistent left superior vena cava, multiple splenunculi in the right hypochondrium, midline liver, bilateral bilobed lungs, a large pulmonary artery (suggestive of severe pulmonary artery hypertension) and a large VSD—a typical constellation of findings described in polysplenia syndrome.

Polysplenia or left isomerism is a rare heterotaxy syndrome characterized by bilateral bilobed lungs, bilateral pulmonary atria, a symmetrical midline liver, and multiple aberrant splenic nodules. The spleen is divided into 2 to 16 masses that are located along the greater curvature of the stomach, either in the right or the left quadrant. The cardiovascular anomalies include left-to-right shunts, partial anomalous pulmonary venous return, and interrupted inferior vena cava (IVC) with azygos or hemiazygos continuation.^1^ We report a case of polysplenia associated with congenital lobar emphysema apart from other typical anomalies—an association that has not been previously reported in the literature.

## CASE

**Figure 1:**
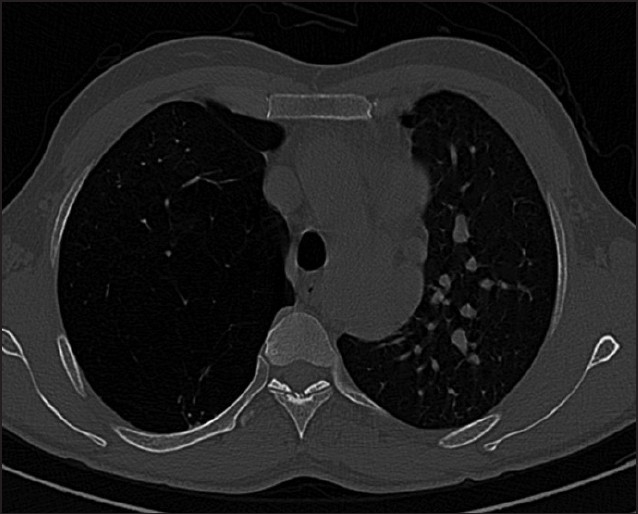
High-resolution CT shows emphysematous right upper lobe with attenuated vascular markings; the accessory hemiazygous is seen draining into the left superior vena cava.

**Figure 2:**
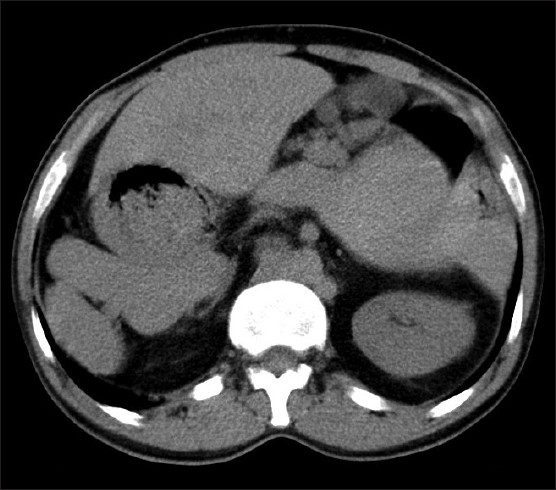
Non-contrast CT of the upper abdomen shows midline liver, stomach on right side of liver and multiple spleens in right hypochondrium.

**Figure 3:**
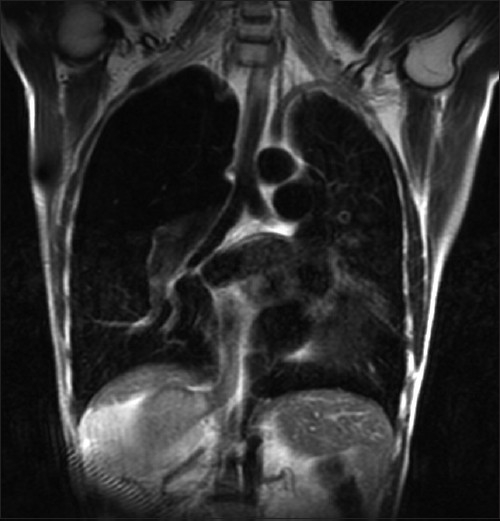
Coronal T2-weighted MRI shows bilateral hyparterial bronchi with emphysematous right upper lobe.

**Figure 4:**
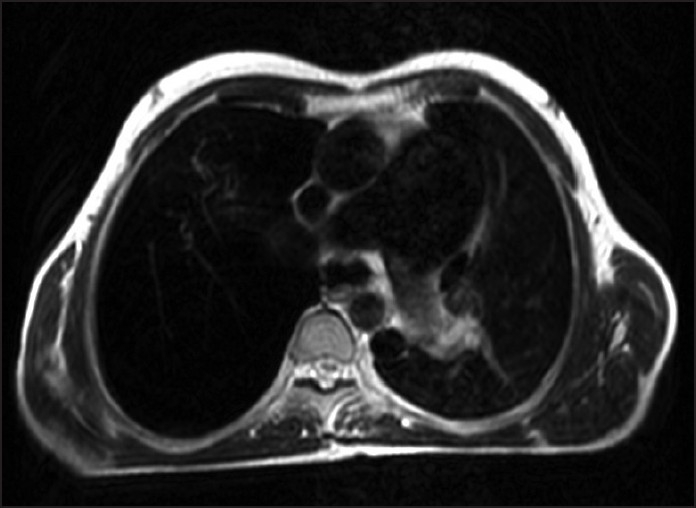
Axial T2-weighted MRI at carinal level shows a grossly enlarged main pulmonary artery, accessory hemiazygous and persistent left superior vena cava.

A 40-year-old man presented with insidious-onset progressive breathlessness. Physical examination showed mild central cyanosis, parasternal heave, a short systolic murmur, and a loud second heart sound. A chest radiograph revealed an enlarged pulmonary conus and hilar vessels and a radiolucent area in the right upper zone. High-resolution CT of the chest showed an enlarged main and lobar pulmonary arteries (suggestive of pulmonary arterial hypertension), with an expanded hyperlucent right upper lobe, suggestive of congenital lobar emphysema. The azygous and accessory hemiazygos veins were enlarged, with drainage of the hemiazygos into a persistent left superior vena cava (SVC), which drained into the coronary sinus (**Figure 1**). The liver was seen in the midline position with a short segment of intrahepatic IVC. The stomach was in the right upper quadrant with multiple splenic masses in relation to the greater curvature (**Figure 2**). MRI confirmed the presence of bilateral bilobed lungs with hyparterial bronchi and of the accessory hemiazygos continuation of the IVC (**Figures 3**, **4**). Transthoracic and transesophageal echo revealed a large ventricular septal defect (VSD) with pulmonary arterial hypertension, which was suggestive of Eisenmenger syndrome. The patient was referred to a tertiary care cardiothoracic surgery department for further management. To the best of our knowledge this is the first reported case of polysplenia associated with congenital lobar emphysema.

## DISCUSSION

The clinical manifestations in polysplenia vary and sometimes can be mild, with many patients (approximately 10%) surviving into mid-adolescence. No single abnormality is pathognomonic for polysplenia; hence, some authors prefer to use the term heterotaxy, and suggest that the different anatomical abnormalities be mentioned while describing a particular patient.^1^,^2^ The various cardiovascular anomalies that may be encountered include atrial septal defect (ASD) (78%), VSD (63%), partial anomalous pulmonary venous return (39%), transposition of the great arteries (31%), right-sided aortic arch (44%), pulmonary valvular stenosis (23%), and subaortic stenosis (8%).^3^ In addition, azygous continuation of the IVC is seen in 65% of cases and bilateral SVC in approximately 47%.^1^,^3^

The abdominal findings may include midline liver (57%), situs inversus (21%), short pancreas, semiannular pancreas, and preduodenal portal vein.^4^–^7^ Renal agenesis or hypoplasia may also be seen.^4^–^7^ Malrotation, including nonrotation, reverse rotation, and midgut volvulus, is also frequently seen in heterotaxy.^8^,^9^ CT as well as MRI can be used to characterize both the visceral and the cardiovascular anomalies in polysplenia and can provide necessary information for surgical planning.^10^ The antenatal diagnosis of cardiosplenic syndromes is possible by sonography supplemented with dedicated fetal echocardiography and color Doppler and, more recently, by ultrafast fetal MRI. Right isomerism is suggested by the presence of complete atrioventricular septal defect, juxtaposition of the IVC and descending aorta, and viscerocardiac heterotaxy. Similarly, in left isomerism, in addition to viscerocardiac heterotaxy and atrioventricular septal defect, azygous continuation of IVC and congenital heart block is also associated.^11^

The pulmonary manifestations include bilateral bilobed lungs and hyparterial bronchi (58%).^1^,^2^ Our patient had a hyperlucent upper lobe with attenuated vascular markings, which was suggestive of congenital lobar emphysema, an entity not described in polysplenia until now. However, the association of congenital heart disease (patent ductus arteriosus, atrial septal defect, ventricular septal defect, total anomalous pulmonary venous return, Tetrology of Fallot with congenital lobar emphysema is well known^12^,^13^ and is reported to occur in 14% to 50% of cases. Both ultrasound and MRI are useful in the antenatal diagnosis of congenital lobar emphysema as well as of other bronchopulmonary malformations. Ultrasound depicts congenital lobar emphysema as a distended fluid-filled anechoic mass that may decrease in size as the gestation progresses. Fetal MRI reveals the high-signal expanded lobe, with compression of the remaining lung and mediastinal deviation (on T2* GRE sequences).^14^,^15^ In our patient, congenital lobar emphysema must have contributed to the worsening of the pulmonary arterial hypertension and dyspnea. The discovery of a bronchopulmonary malformation, including congenital lobar emphysema, on routine antenatal ultrasound should be followed by dedicated fetal echocardiography and a specific search for viscerocardiac heterotaxy.
